# Selected proceedings of the 2010 Summit on Translational Bioinformatics

**DOI:** 10.1186/1471-2105-11-S9-S1

**Published:** 2010-10-28

**Authors:** Eneida A Mendonça, Peter Tarczy-Hornoch

**Affiliations:** 1 Department of Pediatrics and Computation Institute, The University of Chicago, Chicago, IL, USA; 2 Department of Pediatrics, Department of Medical Education and Biomedical Informatics, University of Washington, Seattle, WA, USA

## Background

The third AMIA Summit on Translational Bioinformatics built on the success of the 2008 and 2009 Summits. The Summit continues to highlight the multidisciplinary nature of this rapidly maturing research field and provides the opportunity to forge new transdisciplinary collaborations as the finest minds of the academia, industry, government and non-profit sector are brought together. The six tracks spanned the range from methods for the analyses of molecular through clinical measurements and informatics methods in genetics discoveries and clinical practice.

1: Informatics Methods for the Integrative Analysis of Molecular and Clinical Measurements

2: Computational Approaches to Finding Molecular Mechanisms and Therapies for Disease

3: Informatics Concepts, Tools, and Techniques to Enable Integrative Translational Bioinformatics Research

4: Relating and Representing Phenotypes and Disease for Translational Bioinformatics Research

5: Informatics Methods Bridging Genetics Discoveries and Clinical Practice

6: Dissecting Disease through the Study of Organisms, Evolution, and Taxonomy

## Overview of the 2010 Summit

The breadth and depth of the Summit continues to grow with twenty-five 90-minute scientific sessions. Original research was presented in forty-nine papers and abstract presentations covering all six tracks and spanning from methods to applications. Ten late breaking sessions covered work in press or published since the 2009 Summit including for example a presentation of the analysis of a full human genome in the clinical context. Six of the seven National Centers for Biomedical Computing presented on the relationship of their work to the themes of the Summit along with five other panels covering topics ranging from CTSAs to the eMERGE network. Four keynote presentations addressed the theme of paths toward genomic medicine. Spyro Mousses of TGen presented on “Using BioIntelligence to Search and Understand Individual Genomes” illustrated the ideas in the context of individualized cancer therapy. Serge Saxonov of 23andMe presented on “Consumers, Genomes and Research” demonstrating how this approach could replicate findings from other studies and contribute to new genomic knowledge. Andrew Kasarskis of Sage Bionetwork presented on “Getting from Heterogeneous Data to Actionable Shared Models of Biology” and how these approaches can advance our fundamental understand of the underlying biology. Finally in what has become an annual tradition Russ Altman of Stanford presented a recap of some key developments and papers in his “Translational Bioinformatics Year in Review”.

Slides from selected tutorials, papers, and panels are publicly available at http://summit2010.amia.org/session-details.

## Summary of selected contributions

The eleven papers selected for this supplement to *BMC Bioinformatics* are extended and improved versions of the best papers accepted to the 2010 Summit on Translational Bioinformatics. These papers were nominated by members of the Scientific Program Committee and the Track Chairs and then subject to revision and additional peer review in collaboration with *BMC Bioinformatics*. These papers illustrate the span of the Summit and range from bioinformatics approaches to new knowledge discovery to informatics approaches to foster the application of this type of knowledge. In the following paragraphs we present an overview of the papers in this special issue and their organization.

We start with three papers that describe original informatics methods for the integrative analysis of molecular and clinical measurements. The first paper deals with representing temporal processes in biology, while the other two deal with reasoning across molecular and clinical measurements. Chang and colleagues [1] describe a methodology to identify expression quantitative trait loci (eQTLs) using an information theory approach. The method is used to create transcriptional information maps (TIM). The authors illustrated by constructing a TIM of pediatric lymphoblastic leukemia. Payne et al. [2] reports on an integrative approach that bridges networks of bio-molecular and phenotypic networks with diagnose and treatment of diseases. The article presents a series of experiments that utilize multi-modal approaches to network induction. The authors performed the experiments using data from the National Cancer Institute (NCI) funded Chronic Lymphocytic Research Consortium. Based on the experiment results, the authors propose a conceptual model that aims to identify novel and knowledge-anchored biomarker-phenotype complexes. The last article in this group, by Chen et al [3], proposes a vector-based representation of patient clinical biomarkers to search for latent physiological factors that underlie human diseases directly from clinical laboratory data. The authors analyse factors for five chronic conditions and demonstrate that their methodology is able to offer new insights into the pathophysiological basis of human disease.

One article focuses on computational approaches to finding molecular mechanisms and therapies for disease. More specifically, Zhang and collaborators [4] investigate the use of gene co-expression network analysis to identify potential biomarkers for chronic lymphocytic leukemia. The authors utilize microarray datasets corresponding to multiple types of cancer from the Gene Expression Omnibus and the CODENSE algorithm to identify highly connected gene co-expression networks in which ZAP70, a well characterized biomarker for chronic lymphocytic leukemia (CLL). In the experiment, they were able to identify a new set of genes, which are potential CLL prognostic biomarkers.

The next two articles discuss techniques to enable integrative translational research. The paper by Morgan et al. [5] discuss the differences between meta analysis techniques and how these techniques may be used together with real data to understand a biomedical problem with clinical implications. The work focuses on the application of some of the traditional approaches to meta analysis applied to multiplex gene expression data. Their results show that there is a value in doing meta analysis and combining results from different studies. However, not all techniques perform at the same level of improvement. Harpaz and collaborators [6] demonstrate the feasibility of using language processing and mining techniques (association rule mining) to identify multi-item adverse drug event associations in the Food and Drug Administration’s spontaneous adverse event reporting system. The paper discussed the limitations and challenges that can be attributed to both the method and the quality of the data, and the development of a taxonomy based on the associations identified.

The paper that follows is in the area of representing phenotypes and disease for translational bioinformatics research. The study by Lacson et al.[7] assesses the adequacy of documented asthma markers in the Gene Expression Omnibus database (GEO). They report on 918 asthma samples with 20,640 annotated markers. The analyses show a inadequate variable coverage within GEO and a inconsistent use of terms, although the association between variables was adequate.

Two papers focus on the important topic of informatics methods bridging genetics discoveries and clinical practice. Tatonetti and collaborators [8] describe a new method for candidate pharmacogene discovery from pharmacogenomics genome-wide association studies (GWAS). The new method proposes a knowledge integration and SNP aggregation approach for indentifying genes impacting drug response. For the applied domain, application of warfarin dosing, the method was able to improve the results from a GWAS study, identifying a second pharmacogene. In addition, it was able to discriminate between low-dose and high-dose responders. The work serves as a foundation for future predictive pharmacogenomics. The study by Overby et al.[9] focuses on incorporating genomic knowledge into electronic medical records for pharmacogenomic clinical decision support. The authors assess the feasibility of incorporating pharmacogenomic information knowledge resources (e.g., PharmGKB, FDA approved drug labels, biomedical literature) with clinical relevant knowledge to support decision-making. The paper presents an analysis of the availability of clinically relevant knowledge in the selected resources, as well as an analysis of the availability of computable patient data for pharmacogenomics decision-making.

Finally, the last two papers present research work in the area of dissecting disease through the study of organisms, evolution, and taxonomy. The paper by Yang et al. [10] focuses on animal models and human diseases. The authors propose to elucidate stromal microenvironment signals from probes on human arrays unintentionally cross-hybridizing with mouse homologous genes in xenograph tumor models. A novel method for deriving the underlying biology of the tumor stromal microenvironment, called “biological component analysis” is presented and evaluated. The model enriches the gene expression signals in the stromal component separately from the cancer cell component of the tumor xenograft, allowing researchers to better characterize the tumor microenvironment without additional costs. Rouchka and collaborators [11] focus the work on genome sequence repeats and disease. The authors present an analysis of newly released data fro the 1000 Genomes project with the goal to detect previously unreported full length insertions of the retrotransposon LINE-1. The results suggest that the next generation sequencing data can potentially help in the assessment of the amount of heterogeneity with respect to the LINE-1 retrotransposon amongst humans. It also presents new perspectives for testable hypotheses on the impact that this diversity may have on health individuals.

## Competing interests

The authors declare that they have no competing interests.

## Authors' contributions

Both authors wrote and approved the manuscript. Mendonça was a member of the Scientific Program Committee for the 2010 AMIA Summit on Translational Bioinformatics and the Guest Editor for this special issue; Tarczy-Hornoch was the Chair of the Scientific Program Committee for the 2010 AMIA Summit on Translational Bioinformatics.

## Dedication

The authors and AMIA would like to dedicate this special issue to Marco Ramoni who played a vital role in the establishment of the AMIA Summit on Translational Bioinformatics having served as Track Chair for the 2008 and 2009 Summits and having served as a member of the Scientific Program Committee for the 2010 Summit. Prior to his sudden passing on June 8, 2010 Marco was one of the leading lights of the young field of translational bioinformatics. Marco was an Associate Professor of Pediatrics and Medicine at Harvard Medical School and research faculty at Children’s Hospital, Boston and Brigham and Women’s Hospital. Most recently he was the Director, Biomedical Cybernetics Laboratory, Harvard Medical School, Director, NLM Fellowship in Biomedical Informatics, Children's Hospital Informatics Program., and Associate Director of Bioinformatics, Harvard - Partners Center for Genetics and Genomics. He was also the Director of the course “Biomedical Informatics” at the Harvard-MIT Division of Health Sciences and Technology, core faculty of the course Genomic Medicine at Harvard Medical School and a member of the curriculum committee of the Cellular and Molecular Medicine track of the Medical Physics and Medical Engineering graduate program at Harvard-MIT Division of Health Sciences and Technology. He was co-founder of Bayesware LLC, a software company developing machine-learning programs based on Bayesian methods. He received a PhD in Biomedical Engineering and a BA in Philosophy (Epistemology) from the University of Pavia (Italy) and his postdoctoral training from McGill University, Montreal (Canada). He held academic and visiting positions at the University of Massachusetts, the University of London (United Kingdom), the Knowledge Media Institute (United Kingdom), and the University of Geneva (Switzerland). He is author of well over 100 publications in genetics, biomedical informatics, statistics and artificial intelligence.
